# Stability of equol production capability is associated with the diversity of the gut microbiota of the host: a prospective cohort study

**DOI:** 10.1186/s12866-026-04749-7

**Published:** 2026-01-26

**Authors:** Kaori Iino, Chikara Iino, Shuang Song, Maki Sato, Mako Nakamura, Rina Tanabu, Tsuyoshi Higuchi, Yoshinori Tamada, Ken Itoh, Noriaki Sato, Seiya Imoto, Tatsuya Mikami, Koichi Murashita, Yoshihito Yokoyama

**Affiliations:** 1 Department of Obstetrics and Gynecology, Hirosaki University Graduate School of Medicine, 5 Zaifu, Hirosaki, Aomori 036-8562 Japan; 2 Department of Gastroenterology, Hirosaki University Graduate School of Medicine, Hirosaki, Japan; 3 Department of Obstetrics and Gynecology, Shengjing Hospital, China Medical University, Shenyang, China; 4 Department of Disability and Health, Division of Health Sciences, Hirosaki University Graduate School of Medicine, Hirosaki, Japan; 5 Research Center for Health-Medical Data Science, Hirosaki University Graduate School of Medicine, Hirosaki, Japan; 6 Department of Stress Response Science, Biomedical Research Center, Hirosaki University Graduate School of Medicine, Hirosaki, Japan; 7 Division of Health Medical Intelligence, Human Genome Center, The Institute of Medical Science, The University of Tokyo, Tokyo, 108–8639 Japan; 8 Department of Preemptive Medicine, Innovation Center for Health Promotion, Hirosaki University Graduate School of Medicine, Hirosaki, Japan

**Keywords:** Equol production, Gut microbiome, Gut microbiota diversity, Soy isoflavones, Metabolic pathways

## Abstract

**Background:**

Equol, a gut microbiota–derived metabolite of the soy isoflavone daidzein that acts mainly via estrogen receptor β, has been linked to several health benefits, including relief of menopausal symptoms and prevention of arteriosclerosis. However, little is known about how equol production capability changes over time and how these changes relate to the gut microbiota.

**Methods:**

We analyzed data from 498 community-dwelling adults who participated annually in a health checkup between 2016 and 2019. Equol production was assessed each year using urinary equol and daidzein concentrations. Participants were classified as producers when log10(equol/daidzein) > − 1.75 and were further categorized as stable producers (equol positive in all 4 years), unstable producers (equol positive in at least 1 but not all years), or non-producers (equol negative in all years). Gut microbiota profiles were obtained from fecal samples collected in 2016 and analyzed for bacterial diversity, composition, and predicted metabolic pathways. Dietary daidzein intake was estimated using a validated brief-type self-administered diet history questionnaire.

**Results:**

Of the 498 participants, 30.9% were stable producers, 26.1% unstable producers, and 43.0% non-producers. Stable producers showed significantly higher gut bacterial diversity than unstable producers and non-producers and a distinct microbiota composition, despite comparable daidzein intake across groups. At the genus level, differential abundance analysis indicated that taxa such as *Eubacterium coprostanoligenes* group, *Subdoligranulum*, *Ruminococcus*, *Alistipes*, and *Coprococcus* were enriched in stable producers, whereas *Ruminococcus gnavus* group, *Fusobacterium*, *Eggerthella*, *Flavonifractor*, and *Bacteroides* were more abundant in non-producers. Several genera enriched in stable producers are known short-chain fatty acid producers, whereas some genera enriched in non-producers have been reported in the context of intestinal dysbiosis and inflammation. Functional prediction analysis further indicated that stable producers had microbiota with enhanced pathways related to vitamin B and menaquinone biosynthesis, sulfate degradation, and methanogenesis.

**Conclusions:**

The stability of equol production capability over time is associated with higher gut microbiota diversity and specific compositional and functional features. A gut microbiota composition favoring saccharolytic and butyrogenic bacteria may support sustained equol production, whereas a microbiota dominated by potentially pro-inflammatory taxa may be less conducive to equol production.

**Supplementary Information:**

The online version contains supplementary material available at 10.1186/s12866-026-04749-7.

## Background

Equol originates from soy isoflavones and is transformed by gut microbiota [1]. Among the soy isoflavones and their corresponding metabolites, equol exhibits the most pronounced physiological activity [1]. S-equol functions as a phytoestrogen, primarily exerting its physiological effects through estrogen receptor-β [2, 3]. Marrian and Haslewood identified a compound in 1932 from the urine of pregnant mares, which they named “equol.” This dihydroxyphenol compound signified the initial discovery of equol [4]. In the 1980s, Axelson et al. determined that equol originated from daidzein, a soy component, and is synthesized by specific gut bacteria [5, 6]. As a selective agonist for the estrogen receptor β, equol is hypothesized to have various health benefits [7]. Contemporary research showed that equol not only alleviated menopausal symptoms [8–10] and demonstrated antioxidant capabilities [11], but also exhibited antitumor properties [12, 13]. Moreover, it inhibits the progression of arteriosclerosis-related ailments [14, 15] and non-alcoholic fatty liver disease (NAFLD) [16].

However, differences in the effects of equol and soy isoflavones have been observed between Asian and Western countries [3]. A potential explanation for this discrepancy may lie in the differential equol production capabilities observed between Asian and Western demographics [3, 17].

In East Asia, including Japan, the proportion of equol producers is approximately 50%. In contrast, Western countries exhibit a rate of 20–30% equol producers [3, 17]. This underscores the marked disparity in the prevalence of equol production between Asian nations and their Western counterparts. Additionally, while the daily intake of soy isoflavones in Japan and other East Asian countries ranges from 25 to 50 mg, it is only 2 mg/d in Western countries [18]. In an epidemiological survey conducted in Japan, we observed that 44% of participants were equol producers, and the proportion of these producers notably increased with advancing age [17]. A corresponding age-dependent increase in daidzein intake was observed, suggesting a potential interplay between daidzein consumption and equol production ability. Distinct differences were observed in both α and β diversities between equol producers and non-producers. The α diversity, indicative of the complexity and richness of the microbial community, revealed that equol producers exhibited a greater microbial diversity than non-producers. Interestingly, our study found that most non-producers still harbored equol-producing bacteria, with minimal variance in abundance across most microbial species. Notably, the abundance of the bacterium *Slackia isoflavoniconvertens*, which is significantly more prevalent in equol producers, increased proportionally with age. This suggests that among the equol-producing bacterial species, certain strains might distinctly influence individual equol production capabilities.

Equol is exclusively produced by the gut microbiota and has been shown to be beneficial to human health in multiple studies [3, 5, 7]. Consumers who produce equol may experience various health-promoting effects of equol by increasing their soy product intake [3, 5, 19]. To realize the health benefits attributed to equol without the intervention of supplements, sustained equol production is imperative. However, there is a paucity of epidemiological studies that have assessed equol-producing capabilities longitudinally. Existing literature offers divergent views, with some studies reporting fluctuations in production capacity and others suggesting consistent ability over time [19–21]. In this study, we conducted a longitudinal data analysis among the general population to elucidate how equol-producing capabilities change over time and to identify the characteristics of the gut microbiota that influence variability in equol production.

## Methods

### Study participants

This prospective cohort study evaluated data from the Iwaki Health Promotion Project targeting adult men and women residing in the Iwaki area (Hirosaki, Aomori, Japan). The Iwaki cohort has been studied since 2005 and includes approximately 1000 participants annually, accounting for approximately 10% of the entire Iwaki population. Trained staff documented participants’ height, weight, blood pressure, history of tobacco and alcohol use, and history of illnesses. Blood pressure was measured using standardized methods and body mass index (BMI) was calculated in kg/m^2^. Dietary intake was assessed using the Brief‑type self‑administered Diet History Questionnaire (BDHQ), a validated dietary assessment tool in Japanese adults [22]. Daily daidzein intake (mg/day) was estimated from the BDHQ using the standard nutrient calculation algorithm. In brief, the BDHQ collects frequency and portion size of soy-containing foods over the preceding month, and daidzein intake was calculated by multiplying the reported intake of each food item by its daidzein content based on Japanese food composition tables and summing across all items [22].

**Fig. 1:**
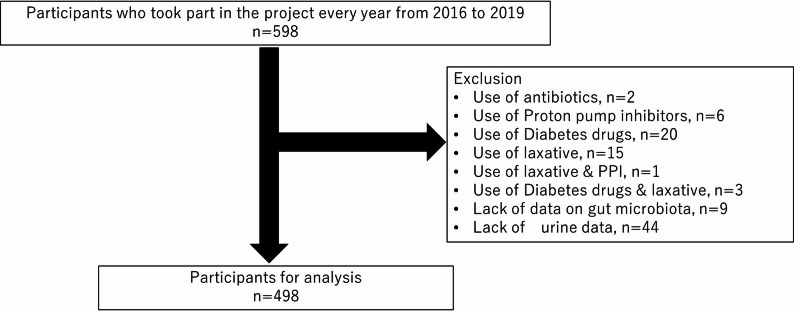
Flow diagram showing participant selection

Initially, 598 individuals who participated annually in the project between 2016 and 2019 were considered. From this group, exclusions were made as follows: two individuals were excluded for antibiotic use, six for proton pump inhibitor use, 20 for diabetes medication use, 15 for laxative use, one for the combined use of laxatives and proton pump inhibitors, three for the combined use of diabetes drugs and laxatives, nine for incomplete gut microbiota data, and 44 for insufficient urine data. Finally, 498 participants were selected for the analysis, as illustrated in Fig. 1. Because this study was nested within the ongoing Iwaki Health Promotion Project, the sample size was determined by the number of eligible participants, and no a priori sample size calculation was performed. This study was approved by the Hirosaki University Graduate School of Medicine Ethics Committee (Approval code; 2016-028-1, 2017-030, 2018-063, 2019-009) and performed in accordance with the 1964 Declaration of Helsinki and its later amendments or comparable ethical standards. All patients provided written informed consent for participation in this study and publication of their data. Clinical trial number: not applicable.

### Defining equol production status

From 2016 to 2019, the concentrations of equol and daidzein in the urine samples collected annually from the participants were measured using a modified high-performance liquid chromatography method. Urine samples were deconjugated by incubating with β-glucuronidase/sulfatase (G0876, Sigma), extracted twice with ethyl acetate, and evaporated. If the log10(equol/daidzein) was > -1.75, the individual was classified as an “equol producer.” Conversely, those with values below this threshold were designated as “non-equol producers.” Based on annual measurements, participants who consistently produced equol were categorized as “stable producers,” those who never produced equol were categorized as “non-producers,” and those whose production capability varied were categorized as “unstable producers.”

### Analysis of gut microbiota

Data on the gut microbiota obtained from the fecal samples collected from participants in 2016 were utilized for the analysis in this study. Fecal samples were collected in commercial containers (TechnoSuruga Laboratory Co., Ltd.,Shizuoka, Japan) and suspended in a guanidine thiocyanate solution (100 mM Tris-HCl [pH 9.0], 40 mM Tris-EDTA [pH 8.0], and 4 M guanidine thiocyanate). These samples were stored at -80 °C until DNA extraction. A series of representative bacterial species in the human gut microbiota were analyzed using primers for the V3–V4 region of 16 S rRNA gene from prokaryotes as described in to previous studies [23, 24]. Sequencing was performed using an Illumina MiSeq system (Illumina, San Diego, CA, USA). The methods for quality filtering of the sequence were as follows: the only reads that had quality value scores of ≥ 20 for > 99% of the sequence were extracted for the analysis. Sequence quality checks, denoising, taxonomic classification, alignment, and phylogenetic tree construction were performed using Quantitative Insights Into Microbial Ecology version 2 (QIIME2) software [25]. Denoising of the sequences was carried out using the DADA2 pipeline, a QIIME2 plugin, to ensure high accuracy in identifying and correcting amplicon errors. This analysis utilized the latest SILVA database release 138 [26] to classify the gut microbiota at the genus level. Intergroup comparisons of microbiota composition were performed using the MaAsLin2 package of R [27], and diversity analysis was performed using QIIME2 [25]. The non-parametric Kruskal-Wallis rank-sum test was used to analyze differences in αdiversity indices (Shannon index) across groups. For β-diversity analysis, the Bray-Curtis distance metric was applied, followed by PERMANOVA analysis to statistically evaluate diversity differences among groups. The results were visualized through two-dimensional principal coordinate analysis (PCoA) plots using the ggplot2 package of R. Functional profiling was performed using PICRUSt2 [28] and visualization was performed using the R package ggpicrust2 [29]. In this functional analysis, predicted pathways were compared only between the stable-producer and non-producer groups, which showed the largest differences in microbiota composition.

### Statistical analysis from epidemiological surveys

Statistical analyses were performed using the R statistical software package. Group comparisons of the epidemiological survey results were performed using Fisher’s exact test, one-way ANOVA, and Tukey’s multiple comparison test. The results of each test are indicated by *p*-values, with values < 0.05 considered statistically significant. All analyses were performed using R version 4.3.2 (R Foundation for Statistical Computing, Vienna, Austria) [30].

## Results

### Baseline characteristics and nutrient intake by Brief-type self-administered Diet History Questionnaire (BDHQ) across three groups classified by equol producer status

There were no significant differences in sex, smoking status, age, BMI, or overall nutrient intake among non-producers, unstable producers, and stable producers of equol. In contrast, daidzein intake differed significantly, being higher in stable producers than in unstable producers, while no significant difference was observed between stable producers and non-producers (Table 1).

**Table 1:** Baseline characteristics and nutrient intake by BDHQ across three groups classified by equol producer status

	Equol producer status			
	Non-producer *N* = 214	Unstable-producer *N* = 130	Stable-producer *N* = 154	*p*.value
Sex (%)				
Man	94	46	61	0.286*
Female	120	46	93	
Smoking (%)				
Never	131	84	97	0.978*
Current	32	18	23	
Past	51	28	23	
Age(Year)	62.38 ± 10.59	64.29 ± 11.47	63.35 ± 11.06	0.287**
Body Mass Index(kg/㎡)	23.11 ± 3.22	22.98 ± 3.16	23.63 ± 3.06	0.170**
Daily Intake Estimated by BDHQ				
Energy (kcal/day)	1921.99 ± 602.13	1825.37 ± 587.73	1907.89 ± 657.11	0.345**
Animal-Based Protein (g/day)	40.73 ± 20.29	37.92 ± 19.49	39.59 ± 18.96	0.438**
Plant-Based Protein (g/day)	30.52 ± 9.80	28.70 ± 9.58	31.47 ± 10.97	0.068**
Animal-Based Fat (g/day)	25.98 ± 12.20	24.09 ± 11.34	24.27 ± 11.53	0.242**
Plant-Based Fat (g/day)	28.17 ± 9.78	26.69 ± 9.66	28.56 ± 11.41	0.277**
Alcohol (g/day)	13.49 ± 21.53	13.18 ± 21.63	13.14 ± 21.06	0.985**
Carbohydrates (g/day)	256.28 ± 87.65	245.12 ± 79.69	256.83 ± 89.44	0.432**
Total Dietary Fiber (g/day)	11.28 ± 4.74	10.40 ± 4.63	11.76 ± 4.73	0.050**
Daidzein (mg/day)	16.02 ± 9.40	14.18 ± 8.94***	17.44 ± 9.74***	0.015**

*BDHQ* Brief-type self-administered Diet History Questionnaire Values are presented as mean ± SD or as percentage

* : By Fisher's exact test

** : By one-way ANOVA

*** : By Tukey’s multiple comparison test

### Analysis of gut microbiota composition

**Fig. 2:**
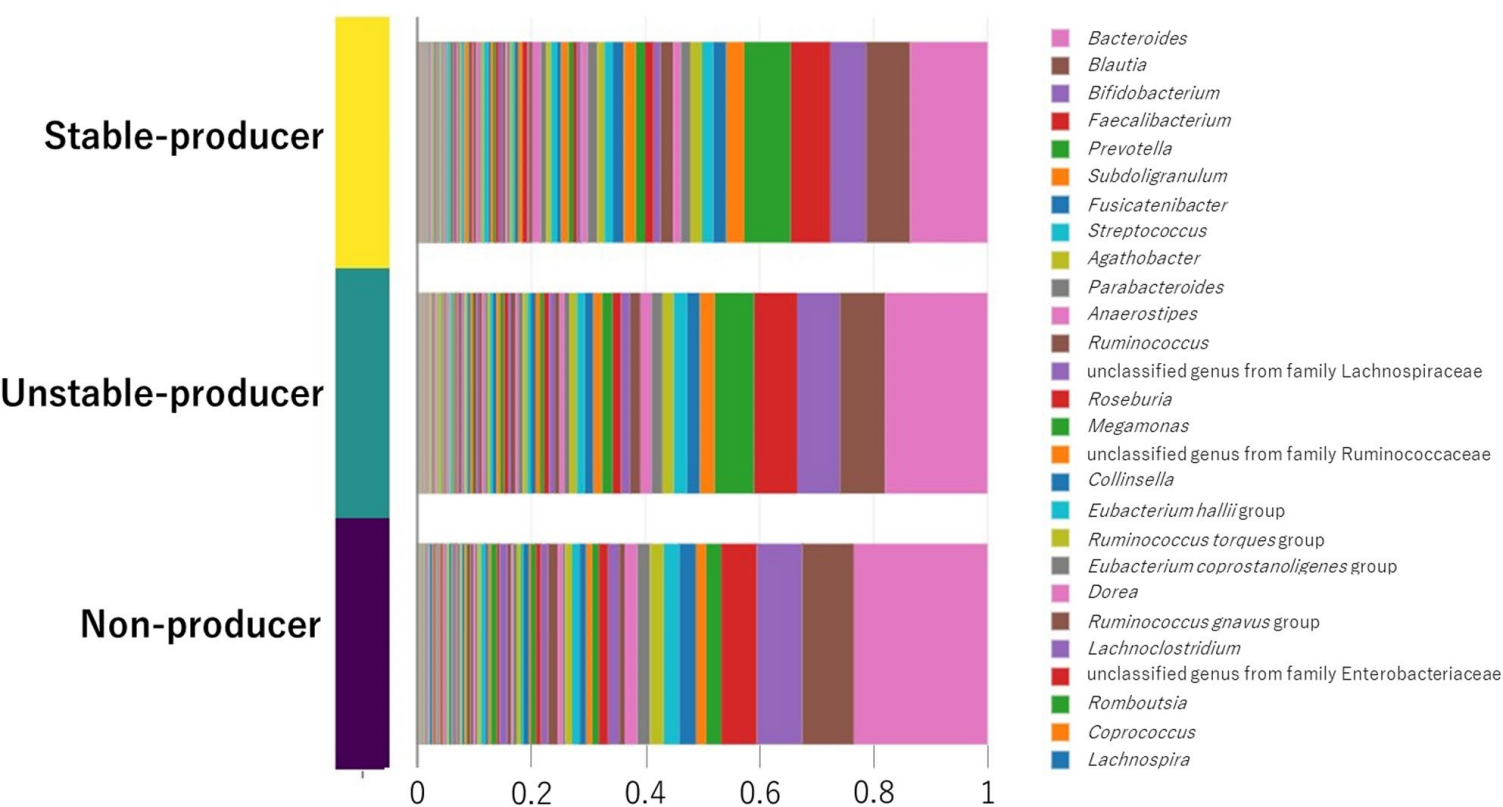
Comparative analysis of gut microbiota genus-level abundance across equol producer groups. Stacked bar charts show the mean relative abundance of predominant bacterial genera in non-producers, unstable producers, and stable producers

**Fig. 3:**
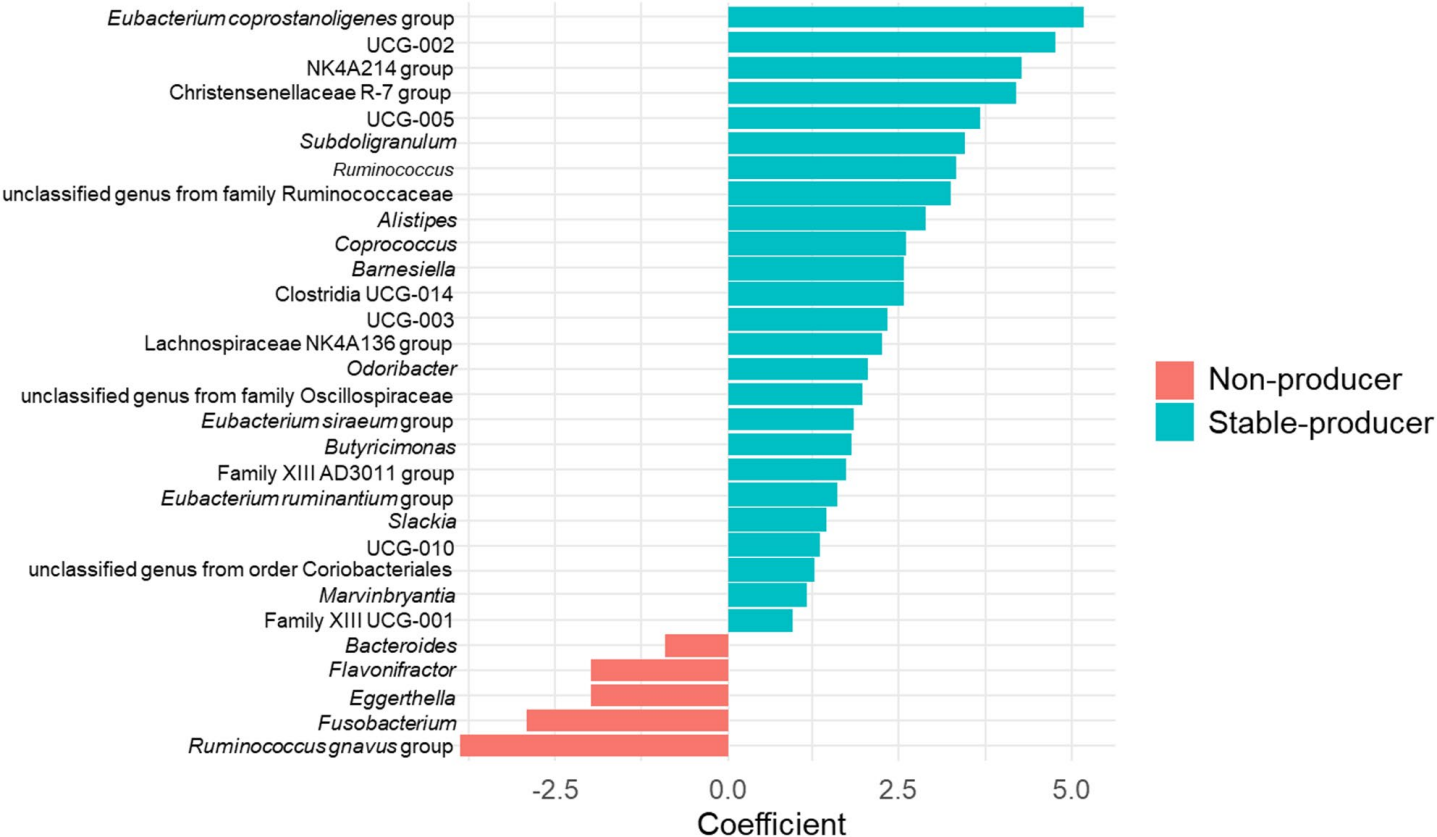
Differentially abundant bacterial genera between equol stable-producers and non-producers. Bacterial genera associated with equol stable-producer status were identified using MaAsLin2, and bars represent regression coefficients, with blue indicating genera enriched in stable-producers and red indicating genera enriched in non-producers; all models were adjusted for age, sex, and BMI

At the genus level, stacked bar plots of predominant taxa (Fig. 2) showed that Bacteroides was more abundant in non-producers, whereas *Butyricimonas* was enriched in stable producers. Overall gut microbiota composition differed among the three equol-status groups. In MaAsLin2 models adjusted for age, sex, and BMI, 115 of the 320 detected genera showed significant differences between producer and non-producer groups (*q* < 0.05). Genera enriched in stable producers included *Eubacterium coprostanoligenes* group, NK4A214 group, Christensenellaceae R-7 group, *Subdoligranulum*, *Ruminococcus*, *Alistipes*, *Coprococcus*, and *Butyricimonas*, whereas *Ruminococcus gnavus* group, *Fusobacterium*, *Eggerthella*, *Flavonifractor*, and *Bacteroides* were more abundant in non-producers (Fig. 3).

### Gut microbiota diversity

**Fig. 4:**
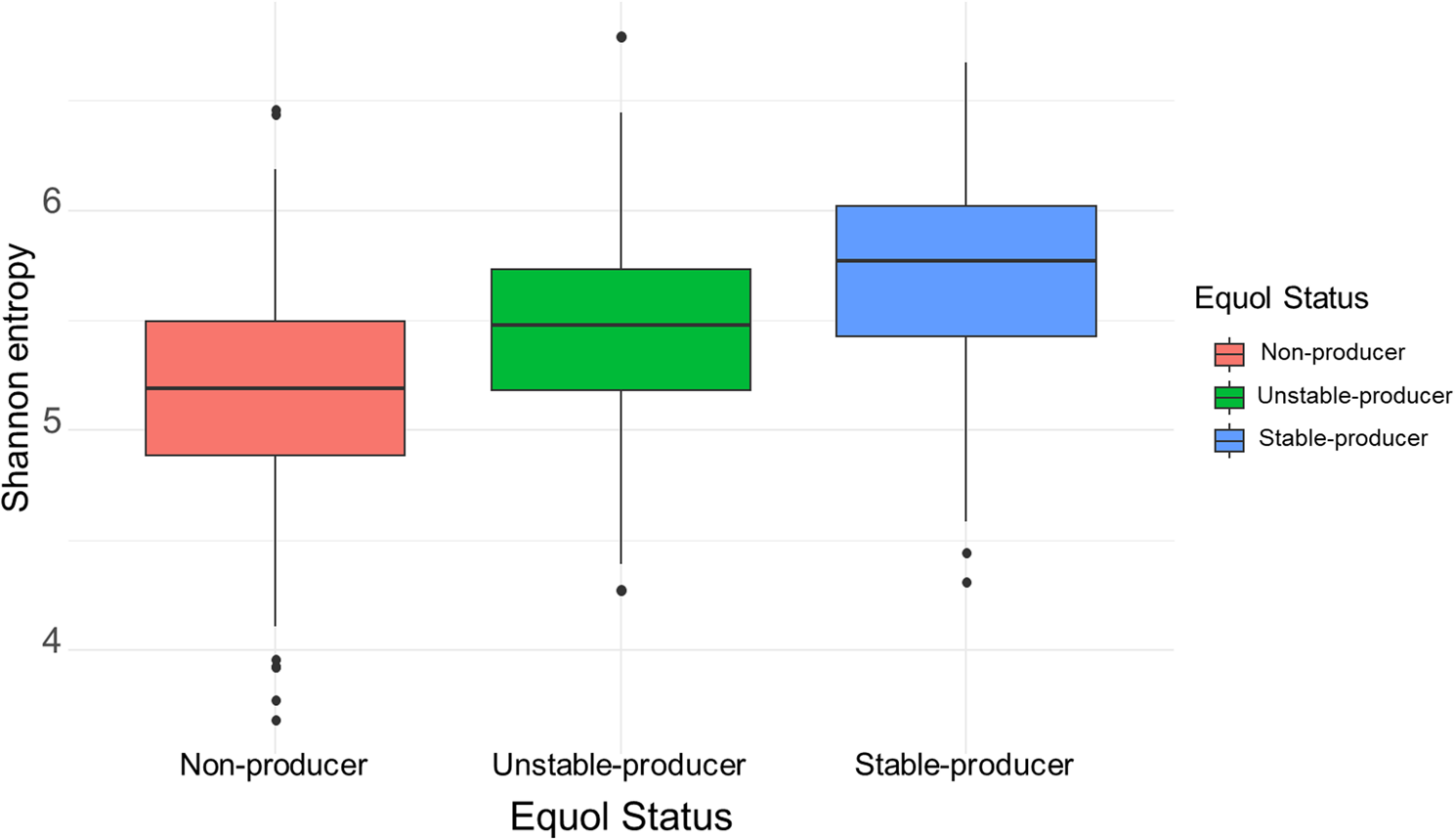
Alpha diversity across equol status groups (Shannon index). Boxplots show Shannon entropy for non-producers, unstable-producers, and stable-producers; the central line indicates the median, the box the interquartile range, the whiskers 1.5× the interquartile range, and dots represent outliers

**Fig. 5:**
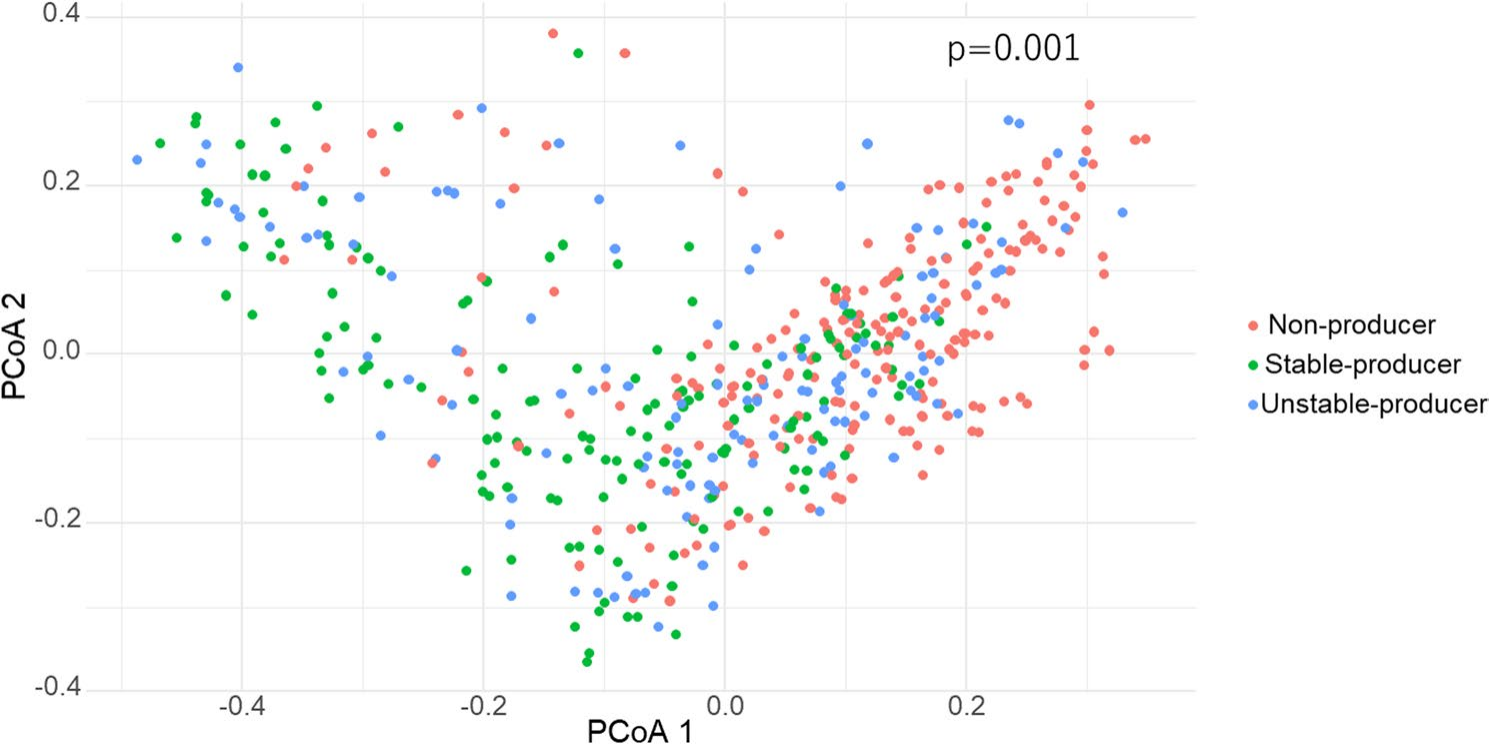
Principal coordinate analysis of gut microbiota by equol-producer status. This plot shows principal coordinate analysis (PCoA) based on Bray–Curtis dissimilarity, where each point represents one participant and colours indicate equol-producer status: non-producers (red), unstable-producers (blue), and stable-producers (green). PCoA1 and PCoA2 are shown on the x- and y-axes, respectively

Pairwise comparisons of the Shannon diversity index showed that microbial diversity was lowest in non-producers, intermediate in unstable producers, and highest in stable producers; all group differences were statistically significant (*q* < 0.001; Fig. 4). Figure 5 shows a principal coordinate analysis (PCoA) based on Bray–Curtis dissimilarity, indicating that gut microbiota composition differed significantly by equol-producer status (PERMANOVA F = 14.92, *p* = 0.001). Pairwise PERMANOVA of Bray–Curtis β-diversity further revealed that stable producers differed significantly from non-producers (R² = 0.033, *q* < 0.001) and from unstable producers (R² = 0.011, *q* < 0.001), and that unstable producers also differed significantly from non-producers (R² = 0.011, *q* < 0.001; Supplementary Table S1).

### Dynamics of gut microbiota function

**Fig. 6:**
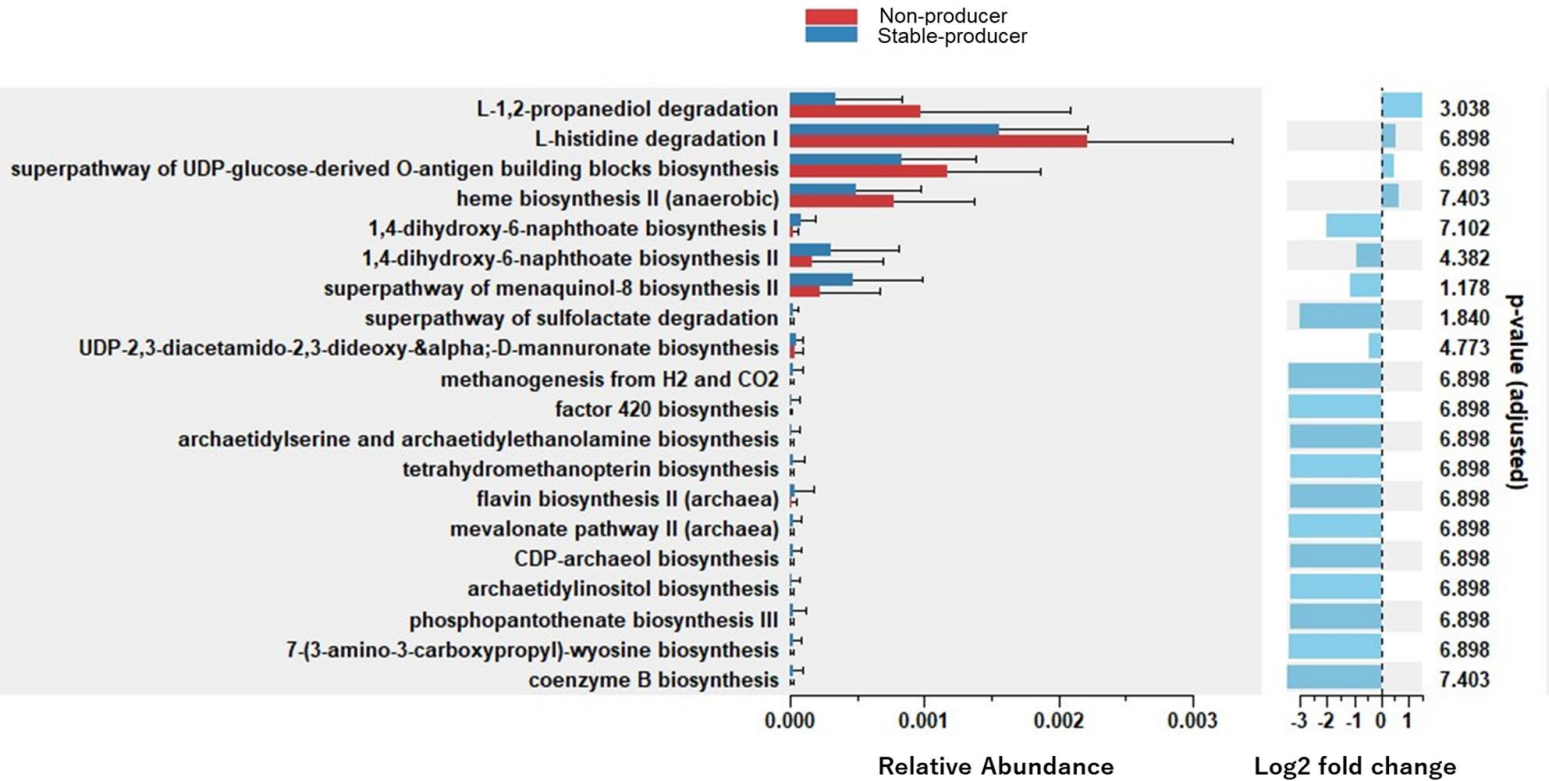
Comparative functional analysis of gut microbiota between equol producers and non-producers. This bar plot shows predicted metabolic pathways that differ between equol stable-producers (blue) and non-producers (red) based on PICRUSt2 analysis; the x-axis represents log₂ fold change in relative abundance, with positive values indicating enrichment in non-producers and negative values indicating enrichment in stable-producers

Functional pathway analysis using PICRUSt2 and ggpicrust2 showed clear differences between equol stable-producers and non-producers (Fig. 6). Non-producers contributed more to L-1,2-propanediol and L-histidine degradation and to the UDP-glucose-derived O-antigen precursor biosynthesis superpathway, whereas stable-producers showed higher contributions to vitamin B and menaquinone biosynthesis, sulfate degradation, and methanogenesis from H₂ and CO₂.

**Fig. 7:**
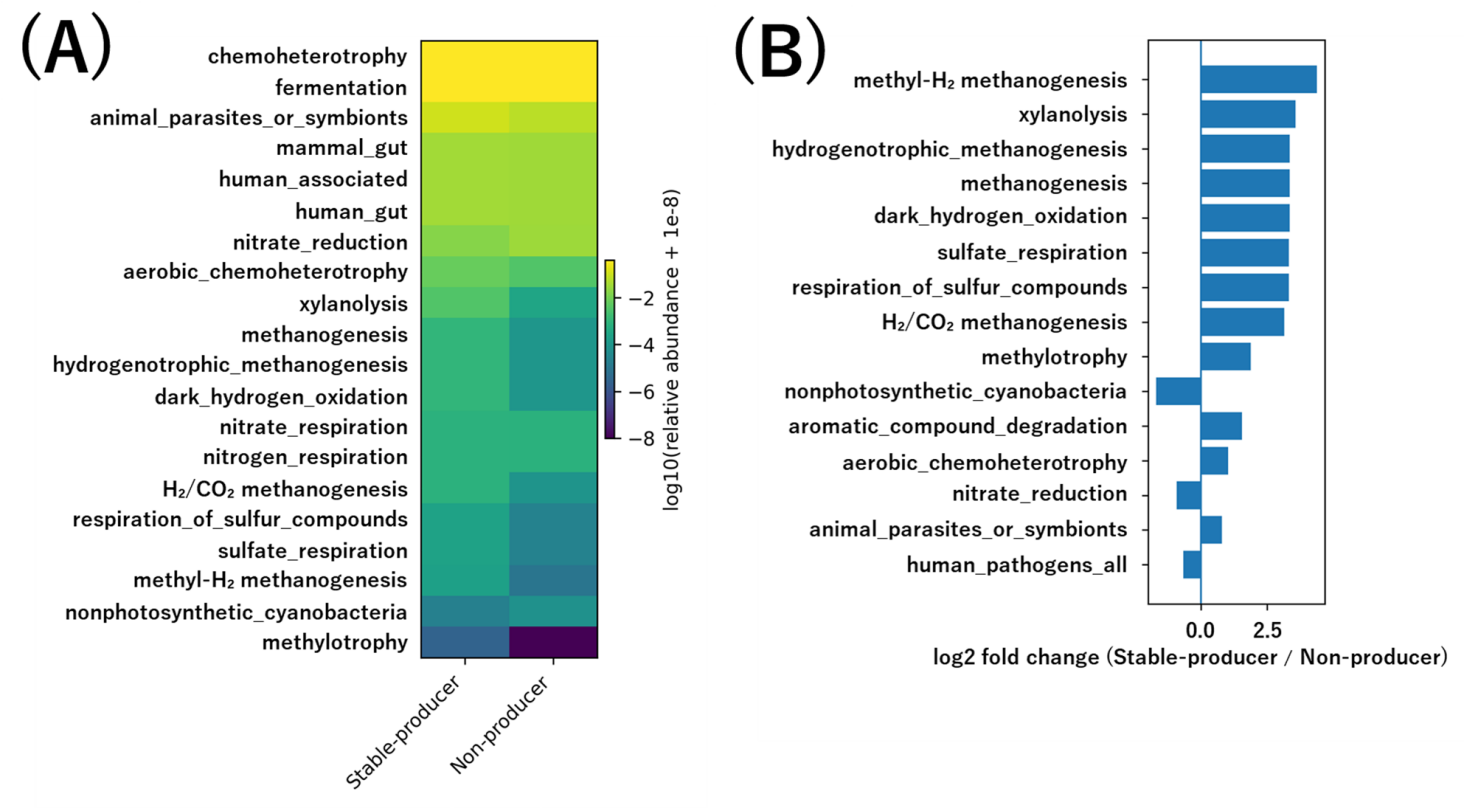
FAPROTAX-based functional profiles of gut microbiota in equol producers and non-producers. (**A**) Group-averaged FAPROTAX functions in equol stable-producers and non-producers. Colors indicate the relative abundance of each functional group (log₁₀-transformed). (**B**) Differentially enriched FAPROTAX functions between equol stable-producers and non-producers, shown as log₂ fold changes. Positive values indicate enrichment in stable-producers, whereas negative values indicate enrichment in non-producers

To further support these functional differences, we performed an additional functional annotation using FAPROTAX, a literature-based taxonomy-to-function mapping approach (Fig. 7). Group-averaged FAPROTAX profiles revealed that the gut microbiota of equol stable-producers was enriched in functions related to fermentation, methanogenesis, and sulfate respiration, whereas non-producers exhibited relatively higher contributions from nitrate-related metabolic functions (Fig. 7A). Consistently, differential analysis based on log₂ fold changes demonstrated an enrichment of anaerobic and fermentation-associated functions in stable-producers compared with non-producers (Fig. 7B).

## Discussion

This study is the first to continuously examine equol-producing capabilities over four years, clarifying changes in these capabilities and revealing specific gut microbiota compositions and metabolic pathways characteristic of equol production. During the observation period, equol producers were divided into three groups: stable, unstable, and non-producers. Baseline surveys showed no significant differences in daidzein intake between stable-producers and non-producers. Gut microbiota analysis revealed that stable-producers had significantly higher bacterial diversity and different compositions than unstable-producers and non-producers. In particular, genus-level differential abundance analysis showed that *Eubacterium coprostanoligenes* group, *Subdoligranulum*, *Ruminococcus*, *Alistipes*, and *Coprococcus* were enriched in stable producers, whereas *Ruminococcus gnavus* group, *Fusobacterium*, *Eggerthella*, *Flavonifractor*, and *Bacteroides* were more abundant in non-producers. Several genera enriched in stable producers include known short-chain fatty acid producers, whereas some genera enriched in non-producers have been reported in the context of intestinal dysbiosis and inflammation [34–39]. These findings suggest that a gut microbiota composition favoring saccharolytic and butyrogenic bacteria may support sustained equol production, whereas a microbiota dominated by potentially pro-inflammatory taxa may be less conducive to equol production. Furthermore, functional analysis of the gut microbiota showed that compared to non-producers, stable-producers had enhanced metabolic pathways, including those for vitamin B synthesis involving iron, menaquinone synthesis, sulfate degradation, and methane production from H_2_ and CO_2_, suggesting enhanced metabolic potential of the gut microbiota.

Although PICRUSt2-based functional prediction does not directly capture the specific enzymes responsible for equol biosynthesis, which depends on reductive enzymes involved in daidzein metabolism, the observed pathway differences likely reflect indirect, community-level metabolic features that support equol production. Pathways related to vitamin B and menaquinone biosynthesis, sulfate reduction, and methanogenesis are characteristic of anaerobic microbial ecosystems and may contribute to maintaining redox balance and cofactor availability favorable for equol-producing bacteria. While some of these pathways were present at relatively low abundance, their consistent enrichment in stable equol producers suggests a shared functional orientation rather than isolated metabolic events.

Consistent with our previous study, equol-producing or equol-associated bacteria were also detectable in non-producers, indicating that equol production capability cannot be explained solely by the presence or absence of specific equol-producing taxa (Iino et al., Nutrients, 2019). The present findings extend this observation by demonstrating that stable equol producers are characterized by higher gut microbiota diversity and a distinct overall community structure, suggesting that sustained equol production depends on a supportive microbial ecosystem rather than on individual taxa alone.

In addition to PICRUSt2-based pathway inference, FAPROTAX analysis provided complementary evidence supporting these functional differences at the level of microbial ecological functions. These predictions should be interpreted as reflecting relative functional tendencies rather than absolute metabolic activity. FAPROTAX revealed that stable equol producers were enriched in functional groups related to anaerobic fermentation, methanogenesis, and sulfate respiration, whereas non-producers exhibited relatively higher contributions from nitrate-related metabolic functions. The concordance between PICRUSt2- and FAPROTAX-based analyses strengthens the interpretation that sustained equol production is associated with a functionally anaerobic and fermentation-oriented gut microbial ecosystem, rather than being an artifact of a single predictive approach.

Equol is a metabolite produced from daidzein by the action of specific gut microbiota and exhibits estrogenic activity because of its structural similarity to 17-β-estradiol [1]. Notably, it does not bind to α estrogen receptors, which are associated with increased risks of breast and endometrial cancers, suggesting that it does not increase the risk for estrogen-related tumors [31]. Its beneficial effects, such as anti-androgenic properties [32], anti-arteriosclerosis activities [14, 15], and potential cognitive enhancement [33], have been documented. Currently, equol-containing supplements are available and people can expect to experience the benefits of equol by consuming these supplements. However, if there is an ability to consistently produce equol, consuming large amounts of soy products can similarly provide the beneficial effects of equol. Previous cross-sectional studies have reported that approximately half of Asians have the ability to produce equol [3, 17]. Our study findings indicate that, when observed continuously, 26% of the equol production capabilities change. When verifying the effect of the presence or absence of equol production capabilities on health, evaluating equol production capabilities multiple times remains advisable. At baseline, the group with stable equol production consumed more daidzein than the group with unstable equol production, indicating that daidzein intake may be involved in stabilizing equol production. However, no difference was observed in daidzein intake between stable producers and non-producers, whereas a clear difference in the gut microbiota composition was observed. Equol stable-producers had higher gut microbiota diversity and more activated metabolic pathways in the gut microbiota than non-producers. Traditionally, equol production is determined by the presence of equol-producing bacteria in the gut [1, 5, 6]. However, our previous study found no significant difference in the abundance of equol-producing bacteria between producers and non-producers [17]. These results suggest that the equol production capability is not only influenced by the presence of equol-producing bacteria but also by the high diversity and abundance of metabolic pathways in the microbiota.

A high diversity of gut bacteria is considered to have various beneficial effects on the human body [34–36]. For example, some bacteria ferment dietary fiber to produce short-chain fatty acids (SCFAs) [36, 37]. The SCFAs have anti-inflammatory and antioxidant properties and are expected to have various health-promoting effects, such as improved digestion and nutrient absorption, regulation of the immune system, and mental health [36, 38]. In this study, *Butyricimonas* was more prevalent in the stable producer group than in the non-producer group. *Butyricimonas* is a bacterium that produces butyrate, an SCFA [39], suggesting that the microbiota of stable producers may have additional beneficial effects on the human body. Our results suggest that the gut microbiota composition of stable equol producers may create a more beneficial intestinal environment for the human body than that of non-producers. Currently, advanced analytical techniques are required to examine individual gut microbiota; however, equol production capability can be easily evaluated using a urine sample, which is more affordable. Equol production may serve as a convenient alternative parameter for assessing individual gut environments. One limitation of this study was the single early morning urine collection used to assess the equol production capability. Because equol is metabolized approximately 8 h after soy product consumption, comprehensive 24-h urine collection would provide a more accurate assessment [40]. However, in the region where our study was conducted, the participants consumed an average of 15.2 mg of soy products per day, suggesting that this factor may not have critically influenced our equol determination outcomes.

## Conclusion

This study showed that the equol production capability changes in some groups. Individual gut microbiota differs significantly in diversity and metabolic pathways based on equol production capability, which may influence the equol production capability. In particular, stable equol producers were characterized by enrichment of several short-chain fatty acid–producing genera and relative depletion of taxa linked to intestinal dysbiosis, suggesting a mechanistic link between a healthier microbial profile and sustained equol production. Furthermore, our results showed that equol producers have higher diversity and abundant metabolic pathways in their gut microbiota. This indicates that the gut environment of equol producers may be beneficial to the human body.

Moreover, the integration of PICRUSt2- and FAPROTAX-based functional analyses consistently indicated that equol stable-producers harbor a gut microbiota enriched in anaerobic, fermentation-associated ecological functions. These findings suggest that equol production capability reflects not only the presence of specific equol-producing bacteria, but also a broader, functionally resilient gut microbial ecosystem, supporting its potential utility as a practical marker of gut microbial health. Table 1 Legend. BDHQ : Brief-type self-administered Diet History Questionnaire. Values are as mean ± SD or as percentage. * : By Fisher’s exact test. ** : By one-way ANOVA. *** : By Tukey’s multiple comparison test.

## Supplementary Information

Supplementary Material 1.

## Data Availability

The raw 16 S rRNA gene amplicon sequences in this study have been deposited with links to BioProject accession number PRJDB35846 in the DDBJ BioProject database.

## Abbreviations

NAFLD: Non-alcoholic fatty liver disease
BMI: Body mass index
QIIME2: Quantitative Insights Into Microbial Ecology version 2
PCoA: Principal coordinate analysis
BDHQ: Brief-type self-administered diet history questionnaire
SCFAs: Short-chain fatty acids
