# Supplementary material for: Stability of equol production capability is associated with the diversity of the gut microbiota of the host: a prospective cohort study
Source: BMC Microbiol. 2026 Jan 26;26:141. doi: 10.1186/s12866-026-04749-7 (PMC12918213; doi:10.1186/s12866-026-04749-7)
Supplement: Supplementary file 1 — Supplementary Material 1. [file 12866_2026_4749_MOESM1_ESM.docx]

Table S1. Pairwise PERMANOVA comparing Bray–Curtis β-diversity among equol status groups.

| **Comparison** | **n₁** | **n₂** | **R²** | **F** | **p-value** | **q-value (FDR)** |
| --- | --- | --- | --- | --- | --- | --- |
| Stable producer vs Non-producer | 154 | 214 | 0.033 | 12.53 | 0.0001 | 0.0001 |
| Stable producer vs Unstable producer | 154 | 130 | 0.011 | 3.13 | 0.0001 | 0.0001 |
| Unstable producer vs Non-producer | 130 | 214 | 0.011 | 3.69 | 0.0001 | 0.0001 |

Table S1. Pairwise PERMANOVA comparing Bray–Curtis β-diversity among equol status groups.

Bray–Curtis distance-based PERMANOVA was used to compare gut microbiota β-diversity between equol status groups. R² indicates the proportion of variance explained by group. p-values were obtained from 9,999 permutations and adjusted for multiple testing using the Benjamini–Hochberg false discovery rate (FDR) procedure.
